# Editorial

**Published:** 1914-11

**Authors:** 


### EDITORIAL

The Business Office of the *Journal-Record* is 23 West Alabama Street.

The Editorial Office is 1014-15 Atlanta National Bank Building.

Address all Business Communications to *Journal-Record of Medicine*, 23 West Alabama Street.

Make remittances payable to *The Journal-Record of Medicine.*

On matters pertaining to the Editorial and Original Communications, address Edgar G. Ballenger, M. D., Atlanta, Ga.

Reprints of Original Articles will be furnished at cost price. Requests for the same should always be made in THE MANUSCRIPT.

We will present, postpaid, on request to each contributor of an original article, twenty (20) marked copies of *The Journal-Record of Medicine* containing such article.

### JOHN BARLEYCORN.

Not long ago, Blythe, in the Saturday Evening Post, gave his opinion of the use of spirituous liquors. It was convincing and showed that it came from a man who knew. Nothing "in the game" could be concealed from him. He had played it all except the tragedy and that he had seen.

Jack London told us about the same time, the graphic story of the tragedy and of escape from it with scars. The same thing is the theme of a story running in a popular magazine at present.

Kansas is sending challenges through its Attorney General to the whole world asking investigation of its claims for increased health and prosperity as they rest upon an actual prohibition of the use of alcohol in the greater part of its territory. The challenge has been accepted and attacks made by the liquor interests and Kansas has refuted the assaults made upon the verity of its claims.

The W. C. T. U. has had its annual meeting and done its best to enter politics with the temperance or rather total abstinence slogan.

But despite it all, the sales of alcohol have not decreased in the country and it would appear that whenever a territory such as Kansas affords went dry, some other territory had to drink the more to keep up the consumption. The people as a whole were not ready for prohibition. In most instances where they have said so as in Georgia and Maine, the prohibitive laws are farces and strong drink is to be had not so much for the asking as just for looking thirsty. The ridiculous "near-beer" rulings of the Georgia magistrates bring the law into contempt and keep it there while drunkards go their way on the streets even more openly than they did in the last years of free sales. Every man knows that "Near Beer" means just the same beer he would get anywhere and that Germany when sending beers to this country has not made a percentage of alcohol to fit Georgia law. Everyone knows that when for instance he goes to the railroad station in Portland and has something in a bottle without a label on it with which to wash down his lobster, he is being served with beer contrary to the law. Every member of a locker club knows as well as he knows anything—as well as he knows how to use sophistry to cover his acts—that he is disobeying the law that was made so stringent that even a physician could not get grain alcohol for his patient without certifying and recording a prescription and having it presented at the drug store by a male adult.

We all know these things and drift along with them in varying degrees of hypocrisy. We have trouble with the chaplains when they protest against this particular kind of lying and unrighteousness if they have nerve enough to speak the truth.

We have needed a lesson. And now we have it. The greatest struggle of opposing might the world has ever seen comes to drag men's minds to facts they would not admit tho' they knew them to be true. The man who drinks alcohol at all is not as good a soldier as the abstainer. National existence is at stake and more dangerously threatened than ever before since we have known about alcohol. Every grain of efficiency is demanded in order to have success. Russia closes down upon the use and manufacture of Vodka absolutely. Drunkenness disappears. France stops Absinthe and the silly mentality it induces is checked.

The Germans drank large amounts of the French wines they captured and they were estopped in their great march toward Paris.

It has taken the killing of thousands every day to teach the powers that be what every intelligent doctor has been teaching or attempting to teach for years—that alcohol decreases efficiency and is not good for human ingestion no matter how much men may like it.

What a stern need it is that makes men see what they are looking at!

But the hundreds of thousands killed will not have died in vain if the intelligent people of the world shall have learned the great medical lesson of the war thus far, and if when peace is settled it shall result in the real and not the hypocritical prohibition of the use of alcohol as a beverage. R. R. D.

